# European intensive care physicians’ experience of infections due to antibiotic-resistant bacteria

**DOI:** 10.1186/s13756-019-0662-8

**Published:** 2020-01-02

**Authors:** Alain Lepape, Astrid Jean, Jan De Waele, Arnaud Friggeri, Anne Savey, Philippe Vanhems, Marie Paule Gustin, Dominique L. Monnet, José Garnacho-Montero, Anke Kohlenberg

**Affiliations:** 1 Clinical Research Unit, Critical care, Lyon-Sud University Hospital, Hospices Civils de Lyon, Lyon, France; 2 Infection Section, European Society of Intensive Care Medicine, Brussels, Belgium; 3 Epidémiologie et Santé Internationale, Centre International de Recherche en Infectiologie (CIRI). Inserm U1111, CNRS UMR5308, ENS de Lyon, Université Claude Bernard Lyon, Lyon, France; 4 Department of Critical Care Medicine, Ghent University Hospital, Ghent, Belgium; 5 European Centre for Disease Prevention and Control (ECDC), Solna, Sweden; 6 Intensive Care Clinical Unit, Virgen Macarena University Hospital, Seville, Spain

**Keywords:** Antimicrobial resistance, Intensive care, Antimicrobial treatment, Third-generation-cephalosporin-resistant Enterobacteriaceae, Carbapenem-resistant Enterobacteriaceae, Meticillin-resistant *Staphylococcus aureus*

## Abstract

**Background:**

Antimicrobial resistance (AMR) compromises the treatment of patients with serious infections in intensive care units (ICUs), and intensive care physicians are increasingly facing patients with bacterial infections with limited or no adequate therapeutic options. A survey was conducted to assess the intensive care physicians’ perception of the AMR situation in the European Union/European Economic Area (EU/EEA).

**Methods:**

Between May and July 2017, physicians working in European ICUs were invited to complete an online questionnaire hosted by the European Society of Intensive Care Medicine. The survey included 20 questions on hospital and ICU characteristics, frequency of infections with multidrug-resistant (MDR) bacteria and relevance of AMR in the respondent’s ICU, management of antimicrobial treatment as well as the use of last-line antibiotics in the six months preceding the survey. For the analysis of regional differences, EU/EEA countries were grouped into the four sub-regions of Eastern, Northern, Southern and Western Europe.

**Results:**

Overall, 1062 responses from four European sub-regions were analysed. Infections with MDR bacteria in their ICU were rated as a major problem by 257 (24.2%), moderate problem by 360 (33.9%) and minor problem by 391 (36.8%) respondents. Third-generation cephalosporin-resistant Enterobacteriaceae were the most frequently encountered MDR bacteria followed by, in order of decreasing frequency, meticillin-resistant *Staphylococcus aureus*, carbapenem-resistant Enterobacteriaceae*,* carbapenem-resistant *Pseudomonas aeruginosa* and vancomycin-resistant enterococci. Perception of the relevance of the AMR problem and the frequency of specific MDR bacteria varied by European sub-region. Bacteria resistant to all or almost all available antibiotics were encountered by 132 (12.4%) respondents. Many physicians reported not having access to specific last-line antibiotics.

**Conclusions:**

The percentage of European ICU physicians perceiving AMR as a substantial problem in their ICU is high with variation by sub-region in line with epidemiological studies. The reports of bacteria resistant to almost all available antibiotics and the limited availability of last-line antibiotics in ICUs in the EU/EEA are of concern.

## Background

Antimicrobial resistance (AMR) is a threat to public health and compromises the treatment of infected patients, in particular the treatment of the most severely ill patients. Intensive care units (ICUs) are often described as the epicenter of AMR and intensive care physicians in Europe are increasingly facing patients infected by bacteria for which limited or no adequate therapeutic options are available [1]. The use of last-line antibiotics has been increasing, including in particular the use of colistin in Southern and Eastern European countries as described by the European Surveillance of Antimicrobial Consumption Network (ESAC-Net) [2]. Only a few new antibiotics have been marketed in recent years and they are not always available for use in ICUs in European Union/European Economic Area (EU/EAA) countries [3].

ARISE (Antimicrobial Resistance in ICU: a Survey in Europe) is a survey of European ICU physicians with respect to their perception of and experience due to infections caused by antibiotic-resistant bacteria. ARISE follows a first survey conducted in 2009 [4] among the members of the European Society of Intensive Care Medicine (ESICM). Since 2009, the AMR situation in Europe has worsened considerably with, for example, increased proportions of third-generation cephalosporin, fluoroquinolone and carbapenem resistance in invasive *Escherichia coli* and *Klebsiella pneumoniae* isolates, even if there has been a stabilization of resistance proportions in recent years [5, 6]. Multiple factors may contribute to the spread of AMR in European countries including cross-border transfer of patients carrying MDR bacteria, transmission of high-risk bacterial clones in and between hospitals and other healthcare settings, overuse and misuse of antimicrobial agents and varying infection control practices and staffing [7–10]. A second survey was therefore conducted in 2017 among physicians working in European ICUs with the aim to determine their current perception of infections due to antibiotic-resistant bacteria and the use of last-line antibiotics.

## Methods

The survey was designed in collaboration with the European Centre for Disease Prevention and Control (ECDC) and reviewed by members of the Infection Section of ESICM. The survey was endorsed by ESICM through its European Critical Care Network in May 2017. It was posted on the ESICM website in the section “Survey of the month” on 19 May 2017 and was closed on 17 July 2017.

### Inclusion criteria

Eligible participants were a convenience sample of physicians in charge of prescribing antibiotics to ICU patients and working in ICUs in the EU/EEA. Physicians outside the EU/EEA could also respond, but their answers were not included in the analysis.

### Dissemination strategy

An invitation to participate was transmitted through ESICM, ECDC and national intensive care societies to their networks via email or web postings. The survey was also promoted via Twitter messages and Facebook posts.

### Survey description

#### Questions on the characteristics of respondents and management of antimicrobial treatment

The ARISE survey included 20 questions. The first questions inquired about the characteristics of the hospital and the ICU in which the respondents worked, as well as their training in intensive care medicine and included the percentage of working time dedicated to the ICU, the frequency of antibiotic prescribing, and the perception of the extent of the problem of AMR. Existence of guidelines on antimicrobial treatment and their origin as well as the availability of local AMR statistics were also explored.

#### Questions on experience with infections due to MDR bacteria

The survey included questions about the number of patients cared for, during the preceding six months, with eight antibiotic-bacteria combinations related to multidrug-resistant (MDR) bacteria: three combinations included Gram-positive bacteria (meticillin-resistant *Staphylococcus aureus* (MRSA), vancomycin-resistant *Enterococcus* spp. (VRE), penicillin-resistant *Streptococcus pneumoniae*) and four combinations included Gram-negative bacteria (third-generation cephalosporin-resistant Enterobacteriaceae, carbapenem-resistant Enterobacteriaceae, carbapenem-resistant *Pseudomonas aeruginosa* and carbapenem-resistant *Acinetobacter* spp.). Participants were invited to evaluate the frequency of encounter with patients with the above mentioned MDR bacteria according to a semi-quantitative scale: encountered very often (> 30 patients), often (11–30 patients), sometimes (3–10 patients), rarely (1–2 patients) and never. Bacteria totally or almost totally resistant to available antibiotics could also be reported.

#### Questions on experience with use of last-line antibiotics

The same approach as for MDR bacteria was applied to the last-line antibiotics used in the participants’ ICUs. A closed list of eight antibiotics (linezolid, daptomycin, fosfomycin, colistin, tigecycline, ceftolozane-tazobactam, ceftazidime-avibactam, and temocillin) was proposed for a similar evaluation of the frequency of prescription by the respondent. The possibility of declaring the unavailability of the above mentioned antibiotics due to either local unavailability (in the hospital/unit) or unavailability in the country was included.

#### Questions on priorities for improvement of the AMR situation in ICUs

Finally, participants were also asked to rank six options having a potential impact on the AMR situation in their ICU by order of priority from 1 to 6, with the option with the lowest number being the highest-ranked option. The options were: “faster/better microbiological diagnostics“, “opportunities for training/education of clinicians for better use of existing antibiotics”, “more resources for infection control“, “more locally adapted guidelines”, “new antibiotics”, and “more opportunity for specialist consultation”. For each option, the ranks were then added and divided by the number of respondents. The original questionnaire is available as supplementary material in Additional file 1.

### Statistical analysis

Statistical analyses were predefined in the statistical analysis plan. EU/EEA countries were grouped in four sub-regions according to the classification used by the Statistics Division of the United Nations: Eastern Europe (Bulgaria, Czech Republic, Hungary, Poland, Romania, Slovakia), Northern Europe (Denmark, Estonia, Finland, Iceland, Ireland, Latvia, Lithuania, Norway, Sweden, United Kingdom), Southern Europe (Croatia, Cyprus, Greece, Italy, Malta, Portugal, Slovenia, Spain) and Western Europe (Austria, Belgium, France, Germany, Liechtenstein, Luxembourg, the Netherlands) [11].

Descriptive statistics were used to summarize characteristics of respondents within the four European sub-regions. The data were compared using the Pearson’s Chi-squared test and the Kruskal-Wallis test.

To improve understanding of factors associated with the perception of the magnitude of the AMR problem by physicians, a multivariate model was constructed including region, type of hospital, number of beds, number of admissions, existence of protocols and access to guidelines as independent variables. All variables were selected based on their significance at a threshold of *p* < 0.05. The multivariate analysis was performed using a cumulative logistic regression with proportional odds (and 95% confidence intervals). Variables for the model were selected following forward regression and validation of the global model was performed using the likelihood ratio test. All analyses were conducted using the R software version 3.4.1.

## Results

Overall, 1141 responses were received, of which 1062 were from physicians working in the EU/EEA and therefore eligible for analysis (Table 1). Nearly half of the responses came from the Western European sub-region, followed by the Southern, Northern and Eastern European sub-region (Table 1). A complete list of the number of respondents by country is available in Additional file 2.

**Table 1:** Characteristics of ARISE survey participants from the EU/EEA, 2017 (*n* = 1062)

	European sub-region								Total		*P*-value
	Eastern		Northern		Southern		Western				
Total replies [n (%)]^a^	94	(8.9)	233	(21.9)	248	(23.4)	487	(45.9)	1062	(100)	
Hospital and ICU characteristics											
Type of hospital [n, (%)]^b^											
General hospital	35	(37.2)	100	(42.9)	124	(50.0)	192	(39.4)	451	(42.5)	< 0.05
University/teaching	59	(62.8)	133	(57.1)	124	(50.0)	295	(60.6)	611	(57.5)	
Type of ICU [n (%)]^b^											
Medical ICU	10	(10.6)	6	(2.6)	22	(8.9)	53	(10.9)	91	(8.6)	< 0.001
Surgical ICU	5	(5.3)	5	(2.1)	12	(4.8)	112	(23.0)	134	(12.6)	
Mixed ICU	66	(70.2)	197	(84.5)	184	(74.2)	289	(59.3)	736	(69.3)	
Other ICU	13	(13.8)	25	(10.7)	30	(12.1)	33	(6.8)	101	(9.5)	
Number of beds in the ICU [Median (IQR)]											
	10	(6–13.5)	12	(8–20)	10	(8–15)	14	(10–20)	12	(8–18)	< 0.001
Number of yearly ICU admissions [n (%)]^b^											
≤ 300	29	(30.9)	15	(6.4)	57	(23.0)	25	(5.1)	126	(11.9)	< 0.001
301–600	44	(46.8)	62	(26.6)	101	(40.7)	160	(32.9)	367	(34.6)	
601–1200	13	(13.8)	101	(43.3)	65	(25.8)	190	(39.0)	368	(34.7)	
> 1200	5	(5.3)	53	(22.7)	22	(8.9)	105	(21.6)	185	(17.4)	
Missing	3	(3.2)	2	(0.9)	4	(1.6)	7	(1.4)	16	(1.5)	
Characteristics of participating ICU physicians											
Training status [n (%)]^b^											
ICU specialist	78	(83.0)	178	(76.4)	208	(83.9)	386	(79.3)	850	(80.0)	< 0.05
Medical specialist	1	(1.1)	5	(2.1)	13	(5.2)	25	(5.1)	44	(4.1)	
Surgery specialist	0	(0.0)	0	(0.0)	0	(0.0)	2	(0.4)	2	(0.2)	
In training for ICU specialist	9	(9.6)	35	(15.0)	16	(6.5)	40	(8.2)	100	(9.4)	
Other	1	(1.1)	7	(3.0)	0	(0.0)	4	(0.8)	12	(1.1)	
Missing	5	(5.3)	8	(3.4)	11	(4.4)	30	(6.2)	54	(5.1)	
Years of clinical practice in ICU [Median (IQR)]											
	20	(11.25–33.5)	15	(8–26)	15	(8–26)	18	(8–35)	17	(8–32.75)	NS
Percentage of time dedicated to ICU [Median (IQR)]											
	61	(51–83.5)	61	(51–81)	96	(71–100)	81	(51–100)	80	(51–100)	< 0.001
Perception of MDR bacteria [n (%)]^b^											
Minor problem	17	(18.1)	115	(49.4)	63	(25.4)	196	(40.2)	391	(36.8)	< 0.001
Moderate problem	34	(36.2)	79	(33.9)	80	(32.3)	167	(34.3)	360	(33.9)	
Major problem	38	(40.4)	31	(13.3)	94	(37.9)	94	(19.3)	257	(24.2)	
Missing	5	(5.3)	8	(3.4)	11	(4.4)	30	(6.2)	54	(5.1)	
Antibiotic therapy prescription [n (%)]^b^											
Commonly	25	(26.6)	60	(25.8)	84	(33.9)	179	(36.8)	348	(32.8)	NS
Often	55	(58.5)	141	(60.5)	140	(56.5)	254	(52.2)	590	(55.6)	
Rarely	14	(14.9)	32	(13.7)	24	(9.7)	54	(11.1)	124	(11.7)	
Management of antimicrobial treatment											
External specialist consultation in case of resistant or difficult to treat infections [n (%)]^b^											
Infectious disease specialist	18	(19.1)	91	(39.1)	140	(56.5)	306	(62.8)	555	(52.3)	< 0.001
Other external specialists	62	(66.0)	133	(57.1)	41	(16.5)	115	(23.6)	351	(33.1)	
No consultation of external specialists	9	(9.6)	1	(0.4)	53	(21.4)	32	(6.6)	95	(8.9)	
Missing/Do not know	5	(5.3)	8	(3.4)	14	(5.6)	34	(7.0)	61	(5.7)	
Availability of protocols for empiric treatment in the ICU [n (%)]^b^											
Yes	61	(64.9)	217	(93.1)	175	(70.6)	311	(63.9)	764	(71.9)	< 0.001
No	28	(29.8)	6	(2.6)	58	(23.4)	138	(28.3)	230	(21.7)	
Do not know	0	(0.0)	1	(0.4)	2	(0.8)	5	(1.0)	8	(0.8)	
Missing	5	(5.3)	9	(3.9)	13	(5.2)	33	(6.8)	60	(5.6)	
Type of guidelines/protocols/recommendations used for antimicrobial treatment [n (%)]^b^											
ICU-specific	48	(51.1)	117	(50.2)	118	(47.6)	236	(48.5)	519	(48.9)	NS
Other (hospital-specific, national or international)	29	(30.9)	91	(39.1)	84	(33.9)	155	(31.8)	359	(33.8)	
None of the above	3	(3.2)	0	(0.0)	5	(2.0)	13	(2.7)	21	(2.0)	
Do not know	2	(2.1)	1	(0.4)	1	(0.4)	3	(0.6)	7	(0.7)	
Missing	12	(12.8)	24	(10.3)	40	(16.1)	80	(16.4)	156	(14.7)	
Availability of resistance statistics for the ICU [n (%)]^b^											
Regularly	58	(61.7)	111	(47.6)	142	(57.3)	261	(53.6)	572	(53.9)	< 0.001
Irregularly	15	(16.0)	51	(21.9)	46	(18.5)	84	(17.2)	196	(18.5)	
Not at all	7	(7.4)	18	(7.7)	15	(6.0)	36	(7.4)	76	(7.2)	
Do not know	2	(2.1)	29	(12.4)	5	(2.0)	28	(5.7)	64	(6.0)	
Missing	12	(12.8)	24	(10.3)	40	(16.1)	78	(16.0)	154	(14.5)	

*EU/EEA* European Union/European Economic Area; *ICU* Intensive Care Unit; *n* number; *IQR* interquartile range; *MDR* Multi-drug resistance

^a^Percentage of all respondents

^b^Percentage of respondents by European region

### Characteristics of respondents

Respondents were working more frequently in university/teaching hospitals than in general hospitals and most frequently in mixed medical-surgical ICUs (Table 1). The majority of respondents were ICU specialists or physicians in training for this specialty. The responding physicians had been for a median of 17 years in clinical practice and the median percentage of their working time dedicated to intensive care was high (80.0%). The majority of respondents declared that they prescribed antibiotics often or commonly. There were significant regional differences for most hospital, ICU and physician characteristics (Table 1).

### Management of antimicrobial treatment

More than half of the respondents stated that they consulted an infectious disease specialist in case of difficult-to-treat infections (Table 1). Almost three-quarters of respondents reported having protocols for empiric treatment of infections in their ICU. Protocols and guidelines for antibiotic treatment were ICU-specific in about half of the cases. Slightly more than half of the respondents received regular reports of AMR statistics for their ICU. There were regional differences in the availability of protocols for empiric treatment and AMR statistics as well as infectious disease specialist consultations. Infections with MDR bacteria in their ICU were rated as a major problem by 257 (24.2%), a moderate problem by 360 (33.9%) and a minor problem by 391 (36.8%) respondents. The ranking of infections with MDR bacteria differed by region and they were more often considered a major problem by respondents in countries of the Eastern and Southern European sub-regions compared to the Northern and Western sub-regions.

### Experience with infections due to MDR bacteria

**Fig. 1:**
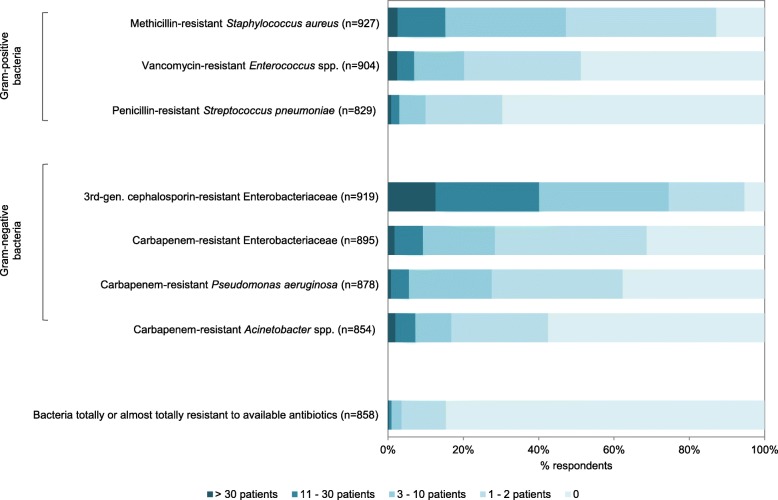
Percentage of ARISE survey participants from the EU/EEA who reported having encountered patients with infections caused by selected antibiotic-resistant bacteria during the past six months prior to the survey (*n* = number of responses for each bacterium)

**Fig. 2:**
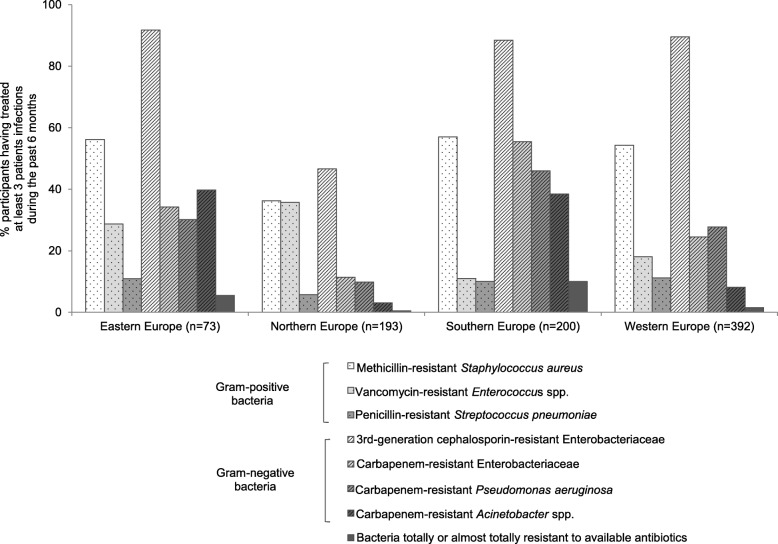
Percentage of ARISE survey participants from the EU/EEA who reported having encountered at least three patients with infections caused by selected antibiotic-resistant bacteria during the past six months, by European region (*n* = 858)

Third-generation cephalosporin-resistant Enterobacteriaceae were the most frequently reported MDR bacteria followed by, in order of decreasing frequency, MRSA, carbapenem-resistant Enterobacteriaceae*,* carbapenem-resistant *Pseudomonas aeruginosa* and VRE (Fig. 1)*.* Bacteria resistant to all or almost all available antibiotics were reported by 132 (12.4%) respondents. Percentages of ICU physicians having cared for three or more patients with MDR bacteria in the past six months are presented by sub-region in Fig. 2 (denominator: 858 respondents after excluding missing or not explicit answers).

### Experience with use of last-line antibiotics

**Fig. 3:**
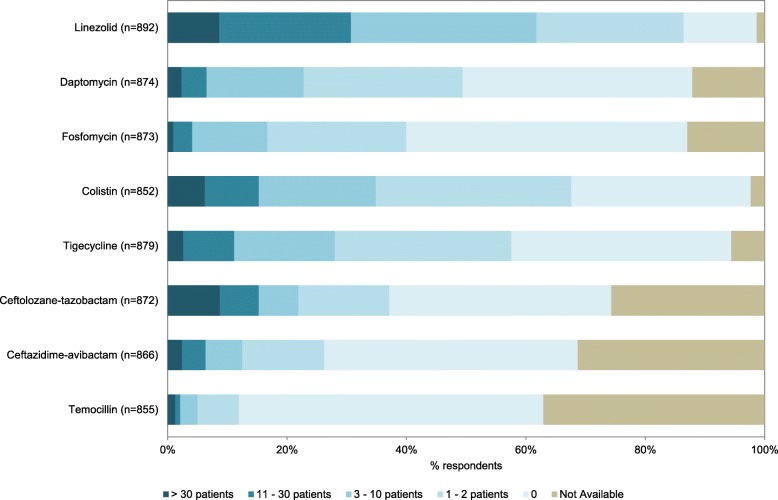
Percentage of ARISE survey participants from the EU/EEA who reported having used the antibiotics listed in the last-line antibiotics list during the past six months (*n* = number of responses for each antibiotic)

Linezolid, colistin, tigecycline and daptomycin were the most frequently prescribed last-line antibiotics (Fig. 3). Many respondents stated that specific last-line antibiotics were not available in their ICU. Unavailability was most frequently the case for an old antibiotic such as temocillin, but the recently marketed antibiotic-beta-lactamase inhibitor combinations such as ceftolozane-tazobactam and ceftazidime-avibactam were also often not available.

### Priorities for improvement of the AMR situation in ICUs

Among six proposed options for improvement of the AMR situation in their ICU, respondents preferred “faster/better microbiological diagnostic methods” with an adjusted score (AS) of 2.3, followed by “training/education of clinicians for better use of antibiotics” (AS = 3.0) and “more resources for infection control” (AS = 3.4). “More locally adapted guidelines” (AS = 3.8), “new antibiotics” (AS = 3.9) and “more opportunity for specialist consultation” (AS = 4.1) were considered as less preferred options. Analysis by European sub-region (data not shown) showed a difference in preferences: “faster/better diagnostic methods” was always the highest ranked option in all four European sub-regions, while “more opportunity for specialist consultation” was classified as the least preferred option in the Southern and Western sub-regions. The need for “new antibiotics” was not classified as the preferred option in any region and was the least preferred option in respondents from the Eastern region.

### Multivariate analysis

The main factor significantly associated with a higher ranking of the AMR problem was the sub-region of origin of the respondent (Table 2). Other significant factors were number of yearly admissions between 601 and 1200 patients, the “other” type of ICU and lack of availability of protocols for empiric treatment in the ICU.

**Table 2:** Multivariate analysis of factors associated with a higher ranking of perception of AMR as a problem among ARISE participants from the EU/EEA

Characteristics	Univariate analysis			Multivariate analysis		
	Odds Ratio	95% CI	*P*-value	Adjusted Odds Ratio	95% CI	*P*-value
European sub-region						
Western	1			1		
Eastern	2.95	1.93–4.53	< 0.001	3.09	1.94–4.92	< 0.001
Northern	0.70	0.51–0.94	< 0.05	0.86	0.62–1.20	NS
Southern	2.33	1.74–3.13	< 0.001	2.67	1.94–3.69	< 0.001
Type of hospital						
General hospital	1			1		
University/teaching	1.30	1.03–1.64	< 0.05	1.29	0.99–1.70	NS (0.06)
Type of ICU						
Medical ICU	1			1		
Surgical ICU	0.75	0.45–1.24	NS	0.92	0.54–1.56	NS
Mixed ICU	0.61	0.40–0.92	< 0.05	0.73	0.47–1.13	NS
Other ICU	0.56	0.32–0.96	< 0.05	0.54	0.30–0.95	< 0.05
Number of beds in the ICU	1.01	1.00–1.02	NS			
Years of clinical practice in ICU	1.00	0.99–1.01	NS			
Number of yearly ICU admissions						
≤ 300	1			1		
301–600	0.76	0.56–1.01	NS	1.05	0.74–1.48	NS
601–1200	1.56	1.21–2.01	< 0.001	1.43	1.10–1.87	< 0.05
> 1200	0.90	0.73–1.11	NS	0.91	0.73–1.13	NS
Training status						
ICU specialist	1					
Other specialty	0.92	0.58–1.46	NS			
Percentage of time dedicated to ICU	1.00	1.00–1.01	NS			
External specialist consultation in case of difficult to treat infections						
Consultation of external specialists	1					
No consultation of external specialists	1.18	0.80–1.74	NS			
Availability of protocols for empiric treatment in the ICU						
Yes	1			1		
No	1.79	1.36–2.35	< 0.001	1.59	1.19–2.13	< 0.05
Type of guidelines/protocols used for antimicrobial treatment						
ICU-specific	1					
Other (hospital-specific, national or international)	1.06	0.83–1.36	NS			
None of these	1.40	0.63–3.12	NS			
Availability of resistance statistics for the ICU						
Regularly	1					
Irregularly	1.19	0.89–1.61	NS			
Not at all	1.04	0.67–1.63	NS			
Do not know	0.71	0.44–1.16	NS			

*CI* confidence interval; *ICU* intensive care unit; *NS* not significant, *p* > 0.05

## Discussion

This survey was completed by 1062 ICU physicians from almost all EU/EEA countries and a majority reported having a moderate to major problem with MDR bacteria in their ICU. ICUs are particularly exposed to AMR. This is due to the specific population of patients cared for in ICUs that accumulate factors known to increase the AMR risk as ICUs combine a high frequency of patients under antimicrobial treatment, a high intensity of care with numerous invasive devices, and a concentration of severely ill patients with multiple previous hospital stays and previous exposures to antibiotics [12]. The substantial proportion (12.4%) of ICU physicians who reported that they had, during the preceding six months, at least one patient with an infection caused by a bacterium resistant to all or almost all antibiotics available in their ICU is of concern. This is an indication that ICU physicians are already encountering situations where options for effective treatment of infections are very limited.

We analyzed the results by European sub-region to take into account that the epidemiology of AMR as described, for example, in the European Antimicrobial Resistance Surveillance-Network (EARS-Net) reports [5], and of antibiotic consumption as reported from ESAC-Net [2] are largely heterogeneous in the EU/EEA. In 2016, EARS-Net reported a wide variation in AMR depending on the bacterial species and the geographical region with higher percentages of AMR being reported by countries in Southern and Eastern Europe. Our multivariate analysis of factors influencing the perception of AMR confirmed the role of the European sub-region, with ICU physicians in Eastern and Southern sub-regions more frequently ranking MDR bacteria in their ICU as a major problem. The other factors significantly associated, in the multivariate analysis, with a higher ranking of perception of AMR as a problem were related to (a) the number of admissions and the type of ICU, which are likely proxies for patient populations with different risks for infection with MDR bacteria, and (b) the lack of protocols for empiric treatment, which might be a sign of the absence of antibiotic stewardship activities that could improve antibiotic prescribing and control of MDR bacteria.

The EARS-Net report also highlighted a high level of resistance to third-generation cephalosporins in Enterobacteriaceae which was reported in our survey as the most frequently encountered type of MDR bacteria in ICUs in the EU/EEA. Our survey also showed that MRSA, despite a decline described in the recent years, is still a problem in ICUs in the EU/EEA. Knowledge of the local resistance situation is beneficial for the correct choice of empiric antibiotic treatment [13]; however, regular resistance statistics were only available in 53.9% of respondents’ ICUs highlighting an important gap.

Responses regarding the use of last-line antibiotics highlighted two important issues. Firstly, it confirmed the lack of availability of older antibiotics in several EU/EEA countries, as already raised by other studies [14, 15]. In particular, colistin and temocillin were not available in all EU/EEA countries [15, 16]. Secondly, our study showed that the recently approved cephalosporin-beta-lactamase inhibitor combinations targeting infections with resistant Gram-negative bacteria: ceftolozane-tazobactam and ceftazidime-avibactam, that received marketing authorization valid throughout the European Union in 2015 and 2016, respectively [17, 18], were not always available in ICUs in EU/EEA countries. However, we were not able to differentiate between non-availability of these antibiotics at national level or at hospital level (e.g. because of cost or organizational reasons). We also did not assess whether availability meant rapid availability from the pharmacy or whether last-line antibiotics had to be ordered from other providers or countries with a delay that may impact appropriate management of severely ill ICU patients. Many respondents mentioned carbapenems in the category ‘other’ last-line antibiotics (data not shown), although they were not included in our list of proposed last-line antibiotics. This is an indication that carbapenems are still considered as last-line antibiotics by many ICU physicians in the EU/EEA.

A previous survey designed by ECDC, the European Medicines Agency and ESICM among European intensive care physicians with partially comparable, but not identical questions and only 95 respondents was published in 2009 [4]. Some results were similar in both surveys. For example, MRSA and third-generation cephalosporin-resistant Enterobacteriaceae were the most frequently reported MDR bacteria in 2009 and in 2017. However, there were differences in the scale used to measure the perception of AMR, which prevents more detailed comparisons. In 2009, 50 (53%) respondents declared having treated at least one patient with a bacterium totally or almost totally resistant to available antibiotics during the preceding six months, which is a higher percentage than the result of the 2017 survey (12.4%). This might be explained by the much lower number of respondents and possible selection bias of these respondents in 2009 as well as possibly by a changing perception, among ICU physicians, of what constitutes “totally or almost totally resistant” and should therefore be interpreted with caution.

To our knowledge, similar European-wide surveys have not recently been performed among ICU physicians. A survey with the same target group, but focusing on the concept of “salvage treatment” and indications for and dosing of specific antibiotics has been conducted by the ESCMID study group for critically ill patients [19]. Other surveys have been conducted in different physician populations or countries, including medical residents of two university hospitals in France and Scotland who were not always working in ICUs or in the position to make decisions about antibiotic treatment [20], physicians of 16 ICUs in the United States with a focus on only Gram-negative MDR bacteria [21], and physicians of only one European country, such as Italy (175 Italian ICUs) [22] or Spain (114 paediatric ICU physicians) [23]. One global survey was investigating specifically the use of polymyxins [24]. Another global survey was conducted among 129 global experts in infections in critically ill patients, among them also 40 European intensivists, as part of development of a global priority pathogen list specific for critical care [25]. Two additional surveys that have been directed specifically to ICU physicians with an important fraction of European respondents investigated the prevention of ventilator-associated pneumonia [26] and central line-associated bloodstream infections [27], but not AMR and antimicrobial use.

Our final question was about ranking options that would have an impact on the AMR in the respondent’s ICU. The preferred option was “faster/better microbiological diagnostic tools”. This is not surprising in the ICU context where the timely replacement of an empiric antibiotic regimen with targeted treatment is of utmost importance. New diagnostic tests are under development, but there might be barriers to their use, often related to costs [28]. Furthermore, the correct use of these new tests is important and, as stated by Messacar et al. [29], diagnostic stewardship is required to direct the appropriate tests to the right patient. “Opportunities for training/education of clinicians for better use of existing antibiotics” was the option ranked second by the respondents, while “new antibiotics” was only ranked fifth suggesting that ICU physicians have more trust in antibiotic stewardship and better targeted use of existing antibiotics than in new antibiotics that might possibly become available in the future [30, 31]. The fact that “more resources for infection control” was ranked third is encouraging, as this indicates that ICU physicians are aware of the close relationship between cross-transmission, ICU acquired-infections and antimicrobial use and that AMR cannot be controlled without the implementation of appropriate infection prevention and control measures and the involvement of ICU physicians in such activities. An interesting example of such involvement is the control of carbapenem-resistant Enterobacteriaceae in Israel by a national intervention [32]. “More opportunity for specialist consultation” was ranked as the least preferred option, indicating that ICU physicians (or at least the respondents to our survey) have sufficient access to infectious disease specialists or consider that their level of competence allows them to manage antimicrobial treatment in severely ill patients.

The strength of this study is its relatively large number of responses from the target group of ICU physicians who are prescribing antibiotics and its ability to provide European sub-region-specific results. This survey has, however, several limitations. Firstly, the results were based on a non-random sample of respondents and a response rate could not be calculated. Given the dissemination strategy, specific categories of ICUs or ICU physicians, especially those with interest in AMR may have been overrepresented. Secondly, we do not know how many ICUs are represented by the respondents as no information allowing the identification of ICUs was collected to maintain the confidentiality of respondents. Thirdly, grouping countries into four regions may mask differences between countries and we were not able to provide results by country due to differences in the number of replies by country. Finally, the main limitation is related to this study being based on a survey asking for individual perception, which cannot be verified or directly compared with epidemiological studies.

## Conclusions

This European survey clearly indicates that (a) European ICU physicians are aware of the threat of AMR, (b) their perception and experience is in line with quantitative data collected in epidemiological studies, (c) infections with bacteria resistant to almost all available antibiotics are already a reality in ICUs in the EU/EEA, and (d) access to last-line antibiotics is sometimes limited. Differences between European sub-regions emphasize the need for national analysis and responses to the local context. At the same time, multinational and multisectoral collaboration is needed to improve microbiological diagnostics and access to antibiotics for ICU physicians, who are on the frontline of the battle against AMR.

## Supplementary information

**Additional file 1.** Questionnaire

**Additional file 2.** Number of respondents by country

## Data Availability

Data can be obtained from ECDC upon request.

## Abbreviations

AMR: Antimicrobial resistance
ARISE: “Antimicrobial resistance in the ICU: a survey in Europe”
EARS-Net: European Antimicrobial Resistance Surveillance Network
ECDC: European Centre for Disease Prevention and Control
ESAC-Net: European Surveillance of Antimicrobial Consumption Network
ESICM: European Society of Intensive Care Medicine
EU/EEA: European Union/European Economic Area
ICU: Intensive care unit
IQR: Interquartile range
MDR: Multidrug-resistant
MRSA: Meticillin-resistant *Staphylococcus aureus*
VRE: Vancomycin-resistant enterococci
