# Supplementary material for: European intensive care physicians’ experience of infections due to antibiotic-resistant bacteria
Source: Antimicrob Resist Infect Control. 2020 Jan 2;9:1. doi: 10.1186/s13756-019-0662-8 (PMC6941296; doi:10.1186/s13756-019-0662-8)
Supplement: Supplementary file 1 — Additional file 1. Questionnaire [file 13756_2019_662_MOESM1_ESM.pdf]

EUROPEAN SOCIETY OF INTENSIVE CARE MEDICINE

The Intensive Connection

Introduction

Dear colleague,

We would like to invite you to take part in the ARISE survey.
ARISE is a survey of European Intensive Care physicians with respect to your experience with infections caused by antibiotic-resistant bacteria
Antibiotic resistance is a threat to public health and compromises the treatment of infected patients, in particular the treatment of the most severely ill patients. Increasingly, intensive care physicians in Europe are confronted with patients infected by bacteria for which limited or no adequate therapeutic options are available.

To obtain information on the perception and experience of European intensive care physicians with infections caused by antibiotic-resistant bacteria, a first survey was conducted in 2009 among the members of the European Society of Intensive Care Medicine (ESICM. The survey was designed by the European Centre for Disease Prevention and Control (ECDC) and EMEA and then proposed to the Scientific Committee of ESICM. The survey was endorsed by ESICM, then posted on the ESICM website in its section “Survey of the month” and published in 2009 (1).

Since 2009, the antimicrobial resistance situation in Europe has evolved. Therefore, a new survey will be conducted among physicians working in European ICUs with the aim to determine the experience of these physicians with infections due to antibiotic-resistant bacteria and with using last-lines antibiotics.

ARISE is a survey of 20 questions. Twelve questions are about your experience with intensive care medicine and antibiotic prescribing, as well as the ICU in which you work. It also includes questions about your perception regarding the problem of antibiotic resistance and the number of patients you have treated, during the preceding six months in the ICU where you work, for infections caused by antibiotic-resistant bacteria.

Welcome to our survey!

The survey should only take 5-10 minutes and your answers will remain completely anonymous.

You can respond once to the survey, and after submission no modification is permitted. Questions with an asterisk (*) are required.

If you have questions about the survey, please contact us at the following addresses: alain.lepape@chu-lyon.fr, astrid.jean@chu-lyon.fr

(1) Lepape A, Monnet DL, participating members, European Society of Intensive Care Medicine. Experience of European intensive care physicians with infections due to antibiotic-resistant bacteria, 2009. Euro Surveill 2009;14(45).

EUROPEAN SOCIETY OF INTENSIVE CARE MEDICINE

The Intensive Connection

SECTION A – Where you work

* 1. In which country do you work?

* 2. What is the type of hospital where you work?

- ( ) General hospital
- ( ) University/teaching hospital

* 3. What is the type of intensive care unit (ICU) where you work?

- Medical
- Surgical
- Mixed
- Neurosurgical
- Coronary
- Burns
- Paediatric
- Neonatal
- Unknown
- Other (please specify)

4. What is the number of beds with mechanical ventilation available in the ICU where you work?

5. What is the number yearly patient admissions in the ICU where you work?

- < 300
- 301-600
- 601-1200
- > 1 200

* 6. How often do you personally prescribe antibiotic therapy to ICU patients?

*if you answer NEVER, the study will be stopped here

- ◯ Commonly (>10 patients per week)
- ◯ Often (3-10 patients per week)
- ◯ Rarely (1-2 patients per week)
- ◯ Never
- ◯ Do not know

SECTION B – About yourself

* 7. What is the status of your training?

- ◯ Intensive care specialist
- ◯ Medical specialist
- ◯ Surgery specialist
- ◯ In training for intensive care specialist
- ◯ In training for any other specialty – Please specify

[ ]

8. How many years of clinical practice do you have in intensive care medicine?

[ ]

9. What is the percentage of time dedicated to caring for the critically ill in your current practice?
in %

[ ]

10. With whom do you consult in the case of resistant or difficult to treat infections in the ICU?

- [ ] Infectious disease specialist
- [ ] Microbiologist
- [ ] Clinical pharmacist
- [ ] I do not consult external specialist (in ICU resources)
- [ ] Do not know
- [ ] Other (please specify)

11. Are there protocols available for empiric antibiotic therapy in the ICU?

- ◯ Yes
- ◯ No
- ◯ Do not know

* 12. Concerning your clinical practice, in the ICU where you work, how would you rate infections due to multidrug-resistant bacteria on the scale below (tick one box) ?

Not a problem										A major problem
◯	◯	◯	◯	◯	◯	◯	◯	◯	◯	◯

SECTION C – Infections due to antibiotic-resistant bacteria

13. During the past 6 months, in the care of how many patients with the following bacteria have you been involved in the ICU?

*Cefotaxime- or ceftazidime- or cetriaxone-resistant

**Imipenem- or meropenem-resistant

	>30 patients (per 6 months)	11-30 patients (per 6 months)	3-10 patients (per 6 months)	1-2 patients (per 6 months)	None (per 6 months)	Do not know
Methicillin-resistant Staphylococcus aureus (MRSA)						
Vancomycin-resistant/intermediate S. aureus (VRSA/VISA)						
Vancomycin-resistant Enterococcus (VRE)						
Penicillin-resistant Streptococcus pneumoniae (PRSP)						
Third-generation cephalosporin*-resistant including ESBL producing Enterobacteriaceae (e.g. Escherichia coli, Klebsiella, Enterobacter)						
Carbapenem**-resistant Enterobacteriaceae (e.g. Escherichia coli, Klebsiella, Enterobacter)						
Carbapenem**-resistant Pseudomonas aeruginosa						
Carbapenem**-resistant Acinetobacter spp.						
Bacteria resistant to all antibiotics tested by your laboratory						
Any other antibiotic-resistant bacteria, not listed above, that posed a problem for patient therapy						

14. If you had any patients with bacteria in the category "bacteria resistant to all antibiotics tested by your laboratory", please specify name(s) of bacteria and resistance

15. If you had any patients with bacteria in the category"Any other antibiotic-resistant bacteria, not listed above, that posed a problem for patient therapy", please specify name(s) of bacteria and resistance

The Intensive Connection

SECTION D - Antibiotics

16. During the past 6 months, in the care of how many patients receiving the below antibiotics have you been involved in the ICU?

	>30 patients (per 6 months)	11-30 patients (per 6 months)	3-10 patients (per 6 months)	1-2 patients (per 6 months)	None (per 6 months)	Not available in my hospital/ICU	Do not know
Colistin	◯	◯	◯	◯	◯	◯	◯
Tigecycline	◯	◯	◯	◯	◯	◯	◯
Linezolid	◯	◯	◯	◯	◯	◯	◯
Ceftolozane/tazobactam	◯	◯	◯	◯	◯	◯	◯
Ceftazidime/avibactam	◯	◯	◯	◯	◯	◯	◯
Daptomycin	◯	◯	◯	◯	◯	◯	◯
Fosfomycin	◯	◯	◯	◯	◯	◯	◯
Temocillin	◯	◯	◯	◯	◯	◯	◯
Any antibiotic, not listed above, that you used to treat antibiotic-resistant bacteria listed in the previous question (see above)	◯	◯	◯	◯	◯	◯	◯

17. If you have marked "Any antibiotic, not listed above, that you used to treat an antibiotic-resistant bacteria listed in the previous question (see above)", please specify name(s) of antibiotic(s)

* 18. To which level are antimicrobial resistance data/statistics available in the ICU?

- ◯ Regularly (at least every 6 or 12 months)
- ◯ Irregularly
- ◯ Not at all
- ◯ Do not know

19. Which guidelines/policies/protocols/recommendations are used for antibiotic therapy in the ICU?

- ☐ ICU-specific
- ☐ Hospital-specific
- ☐ National
- ☐ International
- ☐ None of these
- ☐ Do not know

20. Which of the following options would have an impact on the situation of antimicrobial resistance in your ICU?

Please classify the following options in priority order from 1 to 6 (1: highest priority - 6: lowest priority). Please note that there can be no ex-aequo, each option should have a unique rank attributed.

- [ ] Faster/better microbiological diagnostics
- [ ] More resources for infection control
- [ ] More opportunity for specialist consultation
- [ ] More locally adapted guidelines
- [ ] Opportunities for training/education of clinicians for better use of existing antibiotics
- [ ] New antibiotics

ESICM
EUROPEAN SOCIETY OF INTENSIVE CARE MEDICINE

The Intensive Connection

SECTION E - Comments

21. Please write here if you have comments:
