# Supplementary material for: European intensive care physicians’ experience of infections due to antibiotic-resistant bacteria
Source: Antimicrob Resist Infect Control. 2020 Jan 2;9:1. doi: 10.1186/s13756-019-0662-8 (PMC6941296; doi:10.1186/s13756-019-0662-8)
Supplement: Supplementary file 2 — Additional file 2. Number of respondents by country [file 13756_2019_662_MOESM2_ESM.docx]

**Annex 2 Number of respondents by country**

**Supplemental table 1** Number of respondents from EU/EEA countries

| **Country** | **Respondents (n)** | **%** |
| --- | --- | --- |
| Austria | 47 | 4.4 |
| Belgium | 29 | 2.7 |
| Bulgaria | 1 | 0.1 |
| Croatia | 2 | 0.2 |
| Cyprus | 1 | 0.1 |
| Czech Republic | 10 | 0.9 |
| Denmark | 16 | 1.5 |
| Estonia | 6 | 0.6 |
| Finland | 7 | 0.7 |
| France | 269 | 25.3 |
| Germany | 97 | 9.1 |
| Greece | 33 | 3.1 |
| Hungary | 2 | 0.2 |
| Iceland | 1 | 0.1 |
| Ireland | 10 | 0.9 |
| Italy | 105 | 9.9 |
| Latvia | 0 | 0.0 |
| Liechtenstein | 1 | 0.1 |
| Lithuania | 3 | 0.3 |
| Luxembourg | 6 | 0.6 |
| Malta | 3 | 0.3 |
| Netherlands | 38 | 3.6 |
| Norway | 8 | 0.8 |
| Poland | 56 | 5.3 |
| Portugal | 33 | 3.1 |
| Romania | 5 | 0.5 |
| Slovakia | 20 | 1.9 |
| Slovenia | 13 | 1.2 |
| Spain | 58 | 5.5 |
| Sweden | 9 | 0.8 |
| United Kingdom | 173 | 16.3 |
| **Total EU/EEA countries** | 1062 | 100 |

EU/EEA, European Union/European Economic Area; n, number of respondents.

**Supplemental table 2** Number of respondents from non-EU/EEA countries

| **Country** | **Respondents (n)** | **%** |
| --- | --- | --- |
| Argentina | 3 | 3.9 |
| Australia | 5 | 6.5 |
| Belarus | 1 | 1.3 |
| China | 1 | 1.3 |
| Colombia | 1 | 1.3 |
| Costa Rica | 1 | 1.3 |
| Ecuador | 2 | 2.6 |
| India | 19 | 24.7 |
| Indonesia | 2 | 2.6 |
| Iran | 3 | 3.9 |
| Israel | 2 | 2.6 |
| Japan | 2 | 2.6 |
| Macedonia | 1 | 1.3 |
| Mexico | 3 | 3.9 |
| Pakistan | 1 | 1.3 |
| Peru | 1 | 1.3 |
| Qatar | 1 | 1.3 |
| Russia | 4 | 5.2 |
| Saudi Arabia | 4 | 5.2 |
| Serbia | 3 | 3.9 |
| Sri Lanka | 1 | 1.3 |
| Switzerland | 2 | 2.6 |
| Tunisia | 1 | 1.3 |
| Turkey | 8 | 10.4 |
| Ukraine | 1 | 1.3 |
| United States | 2 | 2.6 |
| Venezuela | 1 | 1.3 |
| Vietnam | 1 | 1.3 |
| **Total Non-EU/EEA countries** | 77 | 100 |
| Country information missing | 2 | NA |

EU/EEA, European Union/European Economic Area; n, number of respondents;

NA, not applicable.
